# Proton beam therapy for pediatric malignancies: a retrospective observational multicenter study in Japan

**DOI:** 10.1002/cam4.743

**Published:** 2016-05-11

**Authors:** Masashi Mizumoto, Shigeyuki Murayama, Tetsuo Akimoto, Yusuke Demizu, Takashi Fukushima, Yuji Ishida, Yoshiko Oshiro, Haruko Numajiri, Hiroshi Fuji, Toshiyuki Okumura, Hiroki Shirato, Hideyuki Sakurai

**Affiliations:** ^1^ Department of Radiation OncologyUniversity of TsukubaTsukubaIbarakiJapan; ^2^ Division of Proton TherapyShizuoka Cancer Center HospitalNagaizumiShizuokaJapan; ^3^ Division of Radiation Oncology and Particle TherapyNational Cancer Center Hospital EastKashiwa, ChibaJapan; ^4^ Department of RadiologyHyogo Ion Beam Medical CenterTatsunoHyogoJapan; ^5^ Department of Child HealthUniversity of TsukubaTsukubaIbarakiJapan; ^6^ Division of PediatricsShizuoka Cancer Center HospitalNagaizumiShizuokaJapan; ^7^ Department of RadiologyNational Center for Child Health and DevelopmentTokyoJapan; ^8^ Department of Radiation OncologyHokkaido University HospitalSapporoJapan

**Keywords:** Observational study, PBT, pediatric, proton beam therapy, radiotherapy

## Abstract

Recent progress in the treatment for pediatric malignancies using a combination of surgery, chemotherapy, and radiotherapy has improved survival. However, late toxicities of radiotherapy are a concern in long‐term survivors. A recent study suggested reduced secondary cancer and other late toxicities after proton beam therapy (PBT) due to dosimetric advantages. In this study, we evaluated the safety and efficacy of PBT for pediatric patients treated in Japan. A retrospective observational study in pediatric patients who received PBT was performed. All patients aged <20 years old who underwent PBT from January 1983 to August 2014 at four sites in Japan were enrolled in the study. There were 343 patients in the study. The median follow‐up periods were 22.6 months (0.4–374.3 months) for all patients and 30.6 months (0.6–374.3 months) for survivors. The estimated 1‐, 3‐, 5‐, and 10‐year survival rates were 82.7% (95% CI: 78.5–87.0%), 67.4% (61.7–73.2%), 61.4% (54.8–67.9%), and 58.7% (51.5–65.9%), respectively. Fifty‐two events of toxicity ≥ grade 2 occurred in 43 patients. Grade 4 toxicities of myelitis, visual loss (two cases), cerebral vascular disease, and tissue necrosis occurred in five patients. This study provides preliminary results for PBT in pediatric patients in Japan. More experience and follow‐up with this technique are required to establish the efficacy of PBT in this patient population.

## Introduction

About 2500 to 3000 new pediatric malignancies are diagnosed every year in Japan. Recent progress using a combination of surgery, chemotherapy, and radiotherapy has improved survival and almost 70% of patients can now be cured 1. However, late toxicities of radiotherapy are a problem in long‐term survivors because children have higher radiation sensitivity and lower radiation tolerance than adults. Reduction in quality of life due to growth and development retardation and secondary cancer is also a significant problem for pediatric patients 2, 3.

The energy of X‐rays commonly used for the treatment of pediatric malignancies reaches a peak at a certain depth and then gradually declines along the irradiation pathway. Therefore, some normal tissue close to the target tumor receives a high dose. Intensity‐modulated radiotherapy (IMRT) has made it possible to irradiate a complex tumor that is difficult to treat with traditional photon radiotherapy, and progress with four‐dimensional radiotherapy now permits precise diagnostic imaging. However, the lower dose area is increased with these techniques and this leads to a significant risk of secondary cancer 4.

In contrast, proton beams have a sharp Bragg peak, with low energy before the peak and almost zero energy after the peak. Therefore, in proton beam therapy (PBT), normal tissue around the tumor receives a reduced dose compared to photon radiotherapy, and this is especially beneficial for pediatric tumors or tumors adjacent to normal tissue for which irradiation should be strictly avoided 5. Recent studies show that PBT can reduce the rate of secondary cancer in long‐term follow‐up 6, 7, 8. In PBT, the tumor control rate is similar to that in photon radiotherapy 9, 10, 11, 12, 13, 14, 15, 16, 17, 18, 19, 20, but late toxicity and the secondary cancer risk should be much lower due to the dose distribution 21. For this reason, PBT has potential as a treatment for pediatric tumors, but fewer institutions have proton beam centers compared to those with normal photon radiotherapy. In this study, we evaluated PBT for pediatric patients in a multicenter study.

## Patients and Methods

A retrospective observational study in pediatric patients who received PBT was performed at four institutions in Japan. All patients aged <20 years old who received PBT at these sites from January 1983 to August 2014 were enrolled without exclusion. The endpoints of the study were safety and efficacy. Data were collected for date of birth, sex, height, weight, disease, reason for PBT, potential for other treatment (photon radiotherapy, surgery), clinical target volume, tumor size, irradiation port number, dose fractionation and treatment period, reference point, combination treatment, sedation, performance status (PS) at the start of treatment, dose to organ at risk, start and end day of PBT, early toxicity, late toxicity, last survival date or date of death, secondary cancer, discontinuation of PBT, and possible comparison of dose distribution to IMRT.

Individual information for each patient was SSL encrypted and anonymized. For patients who were followed at an institution other than that at which PBT was administered, entry into the study was determined based on the rules of the follow‐up hospital or with ethical approval. Survival was analyzed using the Kaplan–Meier method (SPSS, IBM Inc. NY, USA).

## Results

The characteristics of the 343 patients in the study are shown in Table 1. There were 190 males and 153 females, and the median age of all patients was 7 years old (range: 0–19). PS were 0, 1, 2, and unknown for 209, 103, 29, and 2 patients, respectively. PBT was used as a component of initial therapy in 257 patients and for recurrence in 86 patients. Seven patients had multiple primary cancer. Forty‐two patients had received other radiotherapy before PBT and the irradiation field in PBT overlapped with the previous field in 32 of these patients. Surgery was conducted before PBT in 216 patients and after PBT in 7 patients, and 147 patients received concurrent chemotherapy.

**Table 1:** Patients and proton beam therapy characteristics

Age (median)	0–19 (7)
Sex (male/female)	190/153
Disease	
Brain tumor	79
Rhabdomyosarcoma	71
Neuroblastoma	46
Ewing sarcoma	30
Head and neck carcinoma	27
Chordoma	14
Brain stem tumor	17
AVM	8
Others	51
Irradiation site	
Central nervous system	126
Head and neck	105
Abdomen	35
Chest	45
Pelvis	24
Extremities	2
Others	6
PS (0/1/≥2)	209/103/29 (unknown 2)
Initial treatment or recurrence	257/86
Multiple primary cancer	
No/Yes	336/7
Recent irradiation	
No/Yes	301/42^a^
Surgery	
None/preirradiation/postirradiation	120/216/7
Chemotherapy	
None/pre/pre + concurrent/concurrent	72/124/116/31
Total dose (median)	10.8–100 GyE (50.4 GyE)
Combination with photon radiotherapy	
Yes/No	24/319
Target volume	
<100 cc	146
100–500 cc	150
>500 cc	43

AVM, cerebral arteriovenous malformation.

a Fields overlap: 32.

The irradiation dose ranged from 10.8 to 100 GyE (median: 50.4 GyE) and 24 patients received PBT and photon radiotherapy in combination. The irradiation volumes were <100 cc, 100–500 cc, >500 cc, and unknown in 146, 150, 43, and 4 patients, respectively, and 1, 2, 3, and ≥4 ports were used in 51, 147, 57, and 88 patients, respectively. The availability of photon radiotherapy and reasons for performing PBT are shown in Table 2. The information in Table 2 is based on the judgment of radiation oncologists at each facility.

**Table 2:** Availability of photon radiotherapy and purpose of proton beam therapy (PBT)

Availability of photon radiotherapy	
Available, but with increasing risk	285
Unavailable due to critical risk	41
Available with equal risk to PBT	14
Unknown	3
Reason of PBT (multiple selection)	
Reduction of growth retardation	310
Reduction of secondary cancer	298
Overdoses of normal tissues by photon radiotherapy	99
Previous irradiation	25
Patients’ wish	14
Other	3

Photon radiotherapy could not be used in 41 patients because of critical toxicities, and 99 patients received PBT because an adjacent organ could not tolerate photon radiotherapy. The type of treated tumor and the irradiation site are shown in Table 1. Of 41 patients for whom it was considered difficult to perform photon radiotherapy, 3 had late toxicity ≥ grade 2 and 24 died of tumor progression (*n* = 22), secondary malignancy (*n* = 1), and accidental death (*n* = 1).

**Figure 1:**
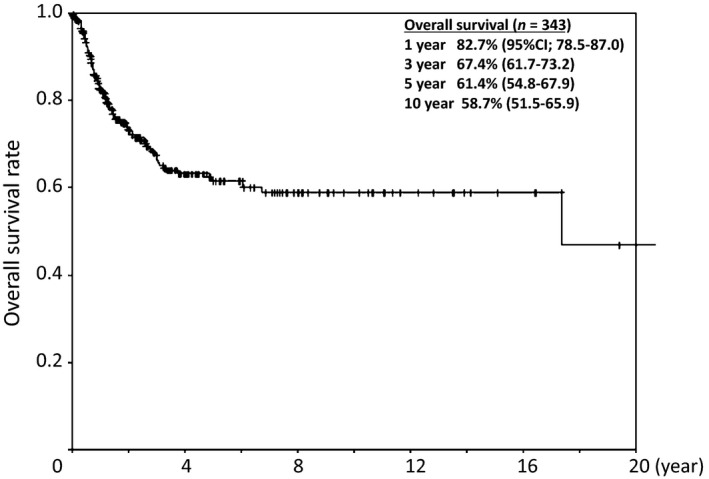
Overall survival curve for all patients.

**Figure 2:**
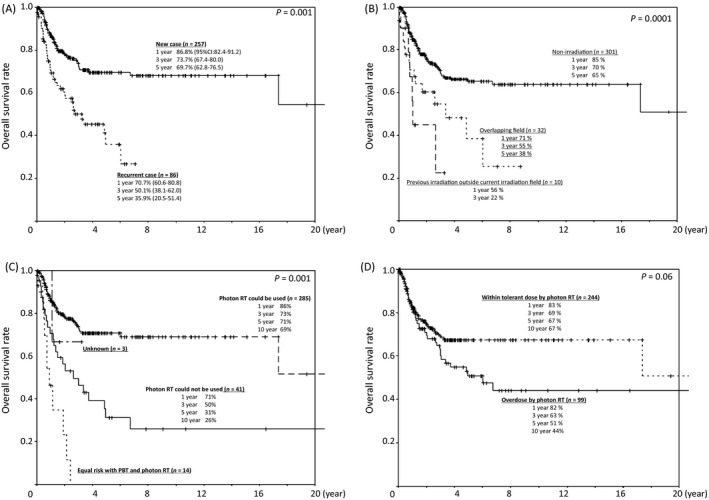
Comparison of overall survival between (A) new and recurrent cases, (B) patients with and without a history of irradiation, (C) patients for whom photon radiotherapy was possible and not possible, and (D) patients with a potential overdose and a tolerable dose in photon radiotherapy. Overall survival was compared, nonirradiation versus previous irradiation (2B), and photon radiotherapy could be used versus photon radiotherapy could not be used (2C).

**Figure 3:**
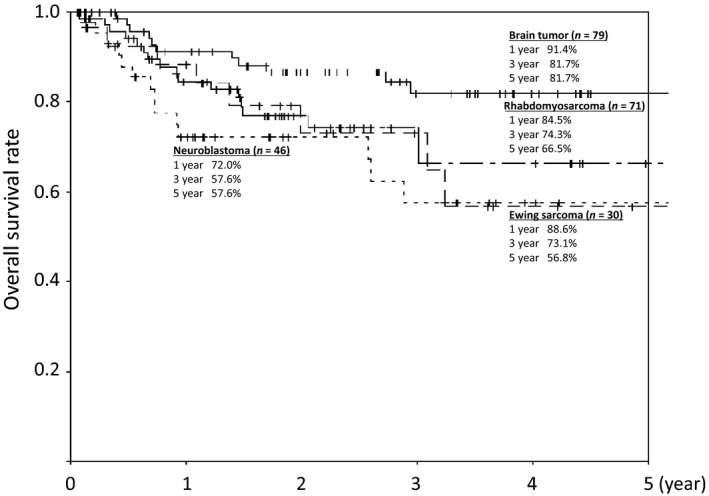
Overall survival curve for brain tumor, rhabdomyosarcoma, neuroblastoma, Ewing sarcoma.

**Figure 4:**
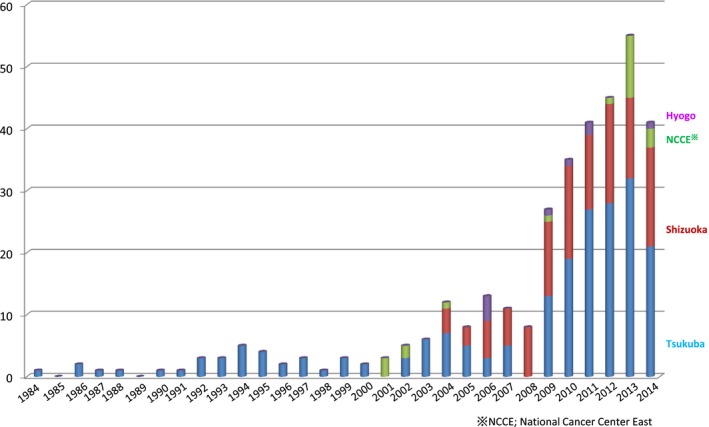
Number of patients treated by proton beam therapy per year.

The median follow‐up periods were 22.6 months (0.4–374.3 months) for all patients and 30.6 months (0.6–374.3 months) for survivors. The estimated 1‐, 3‐, 5‐, and 10‐year survival rates were 82.7% (95% CI: 78.5–87.0%), 67.4% (61.7–73.2%), 61.4% (54.8–67.9%), and 58.7% (51.5–65.9%), respectively (Fig. 1). Overall survival was significantly poorer for recurrent disease (*P* = 0.001; Fig. 2A) and significantly better for patients who had not received previous irradiation (*P* = 0.001; Fig. 2B). Survival was also better for patients who could have received photon radiotherapy instead of PBT compared to those who could not receive photon radiotherapy due to anticipated critical toxicities (*P* = 0.001; Fig. 2C). However, performance of PBT, because the normal tissue dose exceeded a threshold in photon radiotherapy, was not a significant factor influencing survival (*P* = 0.06; Fig. 2D). The estimated 1‐, 3‐, and 5‐year survival rates of brain tumor/rhabdomyosarcoma/neuroblastoma/Ewing sarcoma were 91.4%, 81.7%, and 81.7%/84.5%, 74.3%, and 66.5%/72.0%, 57.6%, and 57.6%/88.6%, 73.1%, and 56.8%, respectively (Figs. 3, 4).

Toxicity events except for secondary cancer are shown in Table 3. There were 52 events of toxicity ≥ grade 2 in 43 patients. Grade 4 toxicities of myelitis, visual loss (2 cases), cerebral vascular disease, and tissue necrosis occurred in five patients, including 3 (60%) who were considered to be unable to tolerate photon radiotherapy (Tables 3 and 4). A secondary tumor developed in seven patients, including two patients with solid malignancies (osteosarcoma and thyroid cancer), four with blood malignancies, and one with benign pituitary adenoma. In‐field tumor development only occurred in the patient with pituitary adenoma (Table 5).

**Table 3:** Toxicity for all patients

Grade	2	3	4
Bone deformity	8	2	0
Growth hormone deficiency	7	1	0
Thyroid dysfunction	7	0	0
Visual/hearing impairment	3	1	2
Brain necrosis/CVD	2	2	1
Gastric/duodenum ulcer	0	1	0
Pneumonitis	0	1	0
Dysphagia	0	1	0
Myelitis	0	0	1
Tissue necrosis	0	0	1

CVD, cerebral vascular disease.

**Table 4:** Grades 3 and 4 toxicities

Grade	Disease	Toxicity	Age/Sex	Dose fractionation irradiation volume (cc)	Previous irradiation	Availability of PRT	Overdoses in case photon RT?
3	Abdominal Ewing	Gastric ulcer	14/F	55.8GyE/31fr >500	No	Available	No
3	RMS	Bone deformity	12/M	39.6GyE/22fr <100	Yes (overlapping +)	Available	No
3	Head and neck cancer	Dysphagia and pneumonitis	16/M	76GyE/38fr 100–500	No	Available	Yes
3	Maxilla osteosarcoma	Oss deformity	10/F	59.4GyE/33fr <100	No	Available	No
3	Brain tumor	Cerebral infarction	3/M	50.4GyE/28fr 100–500	No	Available	No
3	AVM	Brain necrosis	13/F	24GyE/1fr <100	No	Available	No
3	Head and neck cancer	Hearing loss	18/F	72GyE/36fr <100	No	Available	Yes
3	Head and neck cancer	Hearing impairment	2/M	50.4GyE/28fr 100–500	No	Available	Yes
4	Ewing	Myelitis	4/F	55.8GyE/31fr 100–500	No	Available	No
4	Chordoma	Dysopia	18/M	70GyE/25fr <100	Yes (overlapping +)	Available	Yes
4	Head and neck cancer	CVD	16/M	76GyE/38fr 100–500	No	Available	Yes
4	Pelvic osteosarcoma	Tissue necrosis	15/F	70.4GyE/16fr >500	No	Unavailable	Yes
4	Ewing	Dysopia	15/M	59.4GyE/33fr 100–500	No	Available	No

**Table 5:** Secondary tumor

Disease	Age/Sex	Total dose/irradiation volume	Secondary tumor	Outside/inside the irradiation field	Prognosis time from PBT (year)
Head and neck RMS	15/M	60 GyE/100–500 cc	Osteosarcoma	Outside	13.2
Maxillary sinus carcinoma	4/F	40 GyE/<100 cc	Thyroid cancer (papillary carcinoma)	Outside	8.1
Ewing sarcoma	15/F	55.8 GyE/100–500 cc	MDS	–	3.1
Abdominal RMS	1/F	54 GyE/100–500 cc	MDS	–	3
Medulloblastoma	4/M	55.8 GyE/>500 cc	AML	–	1.9
Pelvic RMS	5/M	50.4 GyE/>500 cc	AML	–	1.8
Chordoma	14/F	65 GyE/<100 cc	Pituitary adenoma	Inside	8.8

RMS, rhabdomyosarcoma; MDS, myelodysplastic syndrome; AML, acute myelogenous leukemia; PBT, proton beam therapy.

## Discussion

Reduced toxicity is expected following PBT in pediatric patients because of the favorable dose distribution. In particular, the secondary cancer risk is likely to be much reduced in PBT compared to IMRT because IMRT requires many ports to reach a satisfactory dose distribution and this increases the low dose area. Comparisons of the dose distributions of IMRT and PBT have shown clear advantages of PBT. Hilbrant et al. suggested that the risk of secondary cancer in PBT was half that in IMRT for neuroblastoma and Wilms tumor, and Zhang et al. found that PBT reduces the risk of secondary cancer and cardiac mortality in craniospinal irradiation for medulloblastoma. The lifetime attributed risk for proton/photon therapy ranges from 0.1 to 0.22 and 0.12 to 0.24 for secondary cancer and cardiac mortality, respectively 22. Sethi et al. showed that PBT can reduce the risk of secondary malignancy based on significantly different 10‐year cumulative incidences of RT‐induced or in‐field secondary malignancies of 0% for PBT and 14% for photon radiotherapy in respective follow‐up periods of 6.9 and 13.1 years 22. It should be noted that these periods are still relatively short for this kind of assessment. In our study, 6 (1.7%) of 343 patients had a secondary malignancy, but our follow‐up period is clearly too short for complete evaluation of the risk of secondary malignancy.

In this study, patients who received PBT in 1983 were included, but the median follow‐up periods were only 22.6 months (range: 0.4–374.3 months) for all patients and 30.6 months (range: 0.6–374.3 months) for survivors. This is because the number of patients who could receive PBT was limited in the earlier period of the study, and most patients in the study received PBT recently (Fig. 4). The number of pediatric patients treated by PBT rapidly increased in the 2000s. This change occurred because a central institute for PBT in pediatrics was established in 2002 and a second institute later started for performance of clinical trials of PBT in pediatrics, which has relieved the financial burden on families. Follow up was also difficult for some patients who had to travel long distances due to education or a job. Some patients also do not like to come to the hospital after cure. In addition, photon radiotherapy is covered by insurance, but PBT is expensive in Japan. Also, many severe cases that could not be treated using other therapy were included in this study, and many of these patients could not continue long‐term follow up because of tumor progression, as indicated by the estimated 3‐, 5‐, 10‐year survival rates of 67.4%, 61.4%, and 58.7%, respectively.

The relatively short follow‐up period prevents a firm conclusion on late toxicities. However, our results suggest that PBT may be possible for patients who cannot receive photon radiotherapy, of whom there were 41 in the current study (12%). Treatment outcomes were poor for these patients compared to those for whom photon radiotherapy was possible, and some patients experienced severe toxicities, but at least they had an opportunity for treatment. Recent irradiation history also affected the prognosis, with patients with severe risks or recurrent disease having a poorer prognosis, even with PBT. However, patients who could receive photon radiotherapy but had a risk of toxicity due to an overdose were able to undergo PBT safely. IQ after irradiation may be affected by age at irradiation, dose, irradiation volume, and mean normal brain dose 23. Therefore, PBT may reduce intelligence retardation relative to other radiotherapy. Mizumoto found that PBT for pediatric ependymoma could reduce the dose to normal brain by 28–64% (median 47%) compared to photon radiotherapy 24, and Beltran et al. found that PBT could reduce the whole brain dose by 22% and the whole body dose by 43% in pediatric patients with craniopharyngioma 25. Macdonald et al. showed that normal intelligence was maintained and only a few patients developed evidence of growth hormone deficiency, hypothyroidism, or hearing loss after PBT at doses of 54 to 60 GyE for ependymoma 16. However, it is difficult to demonstrate actual reduction of treatment risk clinically. Pediatric solid malignancies occur less frequently than adult cancer, and a randomized study may be ethically unacceptable because PBT seems to be better in principle. In addition, follow‐up for a few decades is necessary. Further establishment of the efficacy and safety of PBT for pediatric patients will require accumulation of more cases and longer term follow up.

## Conflict of Interest

All authors have no conflicts of interest to disclose.
